# The effect of biological agent on body composition in patients with Crohn’s disease

**DOI:** 10.1186/s12876-023-02742-2

**Published:** 2023-03-30

**Authors:** Eun Jeong Choi, Dong Hoon Baek, Hong Sub Lee, Geun Am Song, Tae Oh Kim, Yong Eun Park, Chang Min Lee, Busan Ulsan Gyeongnam Intestinal Study Group Society (BIGS), Jong Hoon Lee

**Affiliations:** 1 Department of Internal Medicine, Busan Paik Hospital, Inje University College of Medicine, 75 Bokji-Ro, Busanjin-Gu, Busan, 47392 Korea; 2 Department of Internal Medicine, Pusan National University College of Medicine and Biomedical Research Institute, Pusan National University Hospital, Busan, Korea; 3 Department of Internal Medicine, Haeundae Paik Hospital, Inje University College of Medicine, Busan, Korea; 4 Department of Internal Medicine, Gyeongsang National University Hospital, Gyeongsang National University College of Medicine, Jinju, Korea; 5 Department of Internal Medicine, Dong-A University College of Medicine, Busan, Korea

**Keywords:** Crohn's disease, Sarcopenia, Myopenia, Biological products, Body composition

## Abstract

**Background:**

Crohn’s disease (CD) is associated with altered body composition, affecting clinical outcomes. We evaluated the impact of biologics on body composition in CD patients.

**Methods:**

This multicenter longitudinal study across four Korean university hospitals conducted from January 2009 to August 2021 retrospectively reviewed data of CD patients with abdominal computed tomography (CT) before and after the biologic treatment. Skeletal muscle area (SMA), visceral fat area (VFA), and subcutaneous fat area (SFA) of the third lumbar vertebra (L3) on CT were measured. Myopenia was defined as L3 skeletal muscle index (SMI) of < 49 and < 31 cm^2^/m^2^ for men and women, respectively.

**Results:**

Among 112 participants, 79 (70.5%) had myopenia. In the myopenia group, all body composition parameters were significantly increased after the biologic treatment: SMI (37.68 vs. 39.40 cm^2^/m^2^; *P* < 0.001), VFA (26.12 vs. 54.61 cm^2^; *P* < 0.001), SFA (44.29 vs. 82.42 cm^2^; *P* < 0.001), while no significant differences were observed in the non-myopenia group. In multivariate analysis, penetrating CD (hazard ratio, 5.40; *P* = 0.020) was the independent prognostic factor for surgery. Operation-free survival rate tended to decrease in the myopenia group (Log-rank test, *P* = 0.090).

**Conclusions:**

Biological agents can increase all body composition parameters in CD patients with myopenia. These patients are more likely to experience surgery.

**Supplementary Information:**

The online version contains supplementary material available at 10.1186/s12876-023-02742-2.

## Background

Crohn's disease (CD), a chronic inflammatory disease of the gastrointestinal tract, is frequently associated with malnutrition and weight loss and is also accompanied by sarcopenia in 52% of CD patients. [1, 2] Sarcopenia is defined as a syndrome characterized by progressive and generalized loss of skeletal muscle mass plus either low muscle strength or low physical performance, according to the European Working Group on Sarcopenia in Older Persons 2. [3, 4] Sarcopenia, which was considered to be a physical change due to aging, can also occur in young people, reduces the quality of life, and is closely related to mortality. [5, 6] Systemic diseases, such as cancer or autoimmune diseases, can cause sarcopenia. Sarcopenia affect disease prognosis, [7, 8] which lowers an individual’s quality of life and becomes an obstacle to the improvement of the disease, repeating a vicious cycle. Sarcopenia in CD patients affects the clinical outcome of the disease. Sarcopenia is a prognostic factor for intestinal resection in patients with Crohn's disease [9] and increases the risk of postoperative complications. [10] Sarcopenia is associated with loss of response to anti-TNF therapy in CD. [11]

The introduction of biologics has brought major changes to CD patients' quality of life. [12] Biologic agents including infliximab (IFX), adalimumab (ADA), ustekinumab (UST), vedolizumab (VDZ), and so on improved nutritional status, addressing the malnutrition in inflammatory bowel disease (IBD) patients, which can occur in 85% of patients. [13, 14] Moreover, among IBD patients undergoing surgery due to high-dose steroids and complications, the incidence rate of surgery was reduced by 33%–77% with biologic treatment, and long-term remission was maintained. [15, 16]

Computed tomography (CT) can assess myopenia defined as the presence of clinically relevant muscle wasting due to any disease that is not associated with loss of muscle strength or poor physical performance. [17] The previous study showed that the use of infliximab affected the improvement of sarcopenia in Crohn's disease by using various techniques of body composition analysis. [18, 19] However, to our knowledge, there is no study through CT evaluation on the effect of the use of biological agents on body composition changes in Crohn's disease. Therefore, we analyzed the patients’ body composition through CT imaging studies, such as before and after the use of biologics to determine any changes in the parameters. In addition, we evaluated the risk factors associated with surgery after biologic treatment.

## Methods

### Study design

This was a multicenter, longitudinal study of CD patients at four university hospitals in South Korea from January 2009 to August 2021. Eligible patients included those who were at least 18 years of age with a diagnosis of CD, based on clinical, endoscopic, and histopathological criteria. We enrolled patients with moderate-to-severe CD with the indication of biological therapy. Moderate-to-severe CD is defined as the Crohn's Disease Activity Index (CDAI) of 220 or greater. If there is no response to two or more drugs such as corticosteroids or immunosuppressants, or the CDAI of 220 or higher, it is an indication for biologics therapy. [20] The patients underwent abdominal CT within 3 months before starting the biological therapy and follow-up abdominal CT while receiving treatment with biological agents. The exclusion criteria were patients with severe comorbidities, such as cardiovascular disease, chronic renal disease, chronic liver disease, and malignancy.

This study was approved by the Institutional Review Board of Busan Paik Hospital (IRB No. 2021–08-028) and was conducted following the ethical guidelines of the Declaration of Helsinki. The requirement for written informed consent was waived because of the retrospective nature of the study and the analysis used de-identified clinical data.

### Body composition assessment based on CT

**Fig. 1:**
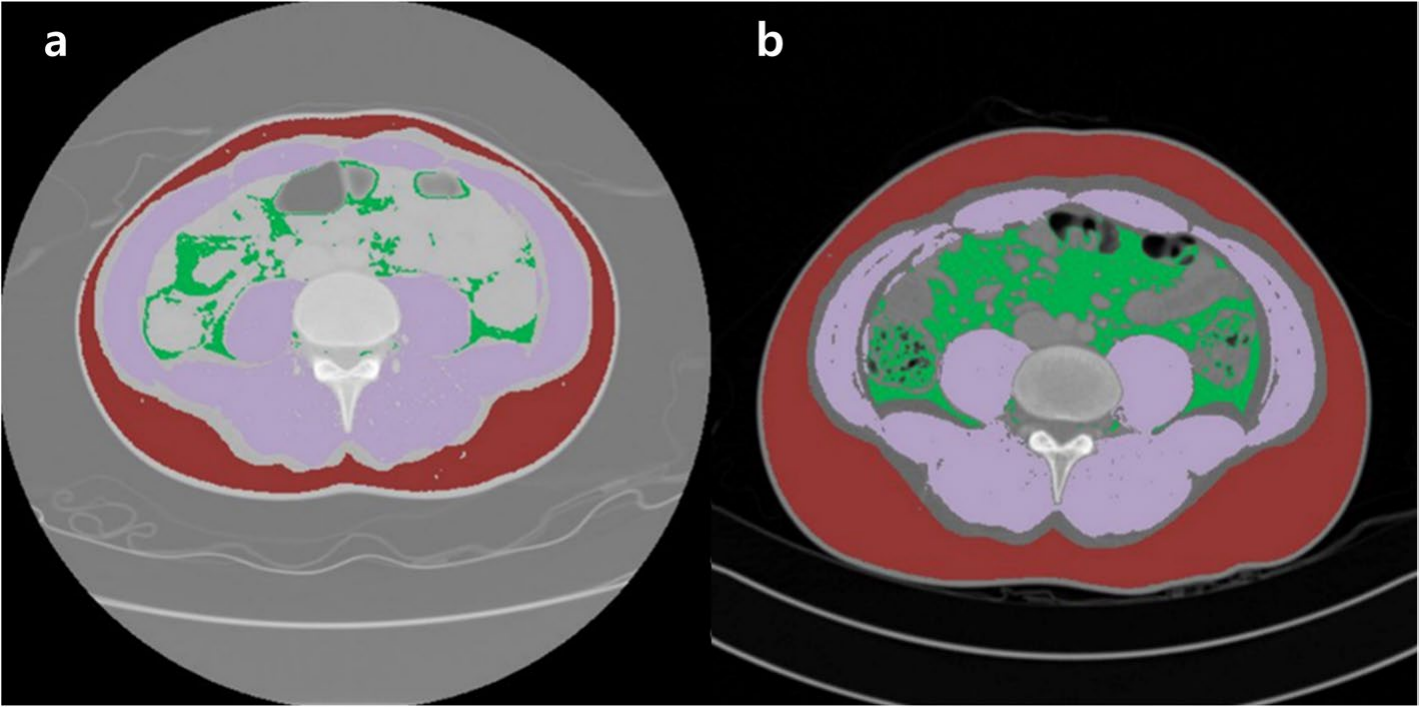
Evaluation of the body composition parameters at the L3 vertebral level using computed tomography scans. (**a**) Baseline (**b**) After treatment with biologic agents. Purple: skeletal muscle area (SMA), red: subcutaneous fat area (SFA), green: visceral fat area (VFA)

The cross-sectional area at the level of the L3 vertebra on CT was selected for the assessment of body composition, such as skeletal muscle area (SMA), visceral fat area (VFA), subcutaneous fat area (SFA), right and left psoas muscle areas (PSAs), total fat area (TFA), and intramuscular fat area (IMFA). The areas of body composition were measured according to predetermined thresholds for the Hounsfield unit on CT using AsanJ-MorphometryTM software based on ImageJ (NIH, Bethesda, MD, USA; Fig. 1; this open-source software is available at https://datasharing.aim-aicro.com/morphometry). The measurements were performed by a researcher blinded to patient information and outcomes. Also, the measurements of body composition were performed according to the instructions of the radiology doctor.

Skeletal muscle index (SMI) was defined as the SMA divided by height in meters squared and used to identify patients with myopenia. The cut-off values of SMI were 49 cm^2^/m^2^ and 31 cm^2^/m.^2^ for Korean men and women, respectively. [21]

### Demographic and clinical parameters

The electronic medical records were used to analyze patient demographics, including age, sex, height, body weight, body mass index (BMI), smoking history, disease characterization, and according to the Montreal classification duration of disease and laboratory parameters, such as serum C-reactive protein (CRP), albumin, and hemoglobin levels. Biological agents included IFX, ADA, UST, and VDZ. More than one biologic was used, as patients may lose their response to some biological agents. Abdominal surgeries associated with CD included colectomy, ileocolonic resection, segmental small bowel resection, and anal fistula operation.

### Statistical analysis

Continuous variables were presented as median (interquartile range) because variables were unevenly distributed. Categorical variables were presented as numbers (percentages). The Chi-square (χ^2^) test was used to compare categorical variables, and Mann–Whitney U test was used for continuous variables. Wilcoxon signed-rank test was used to compare the parameters of the laboratory, including CRP, hemoglobin, albumin, and CT values, including SMA, SMI, PMA, SFA, VFA, TFA, and IMFA before and after the biologic treatment. The correlation between the SMI and clinical variables was analyzed using Spearman's rank correlation. To evaluate the prognostic outcomes of surgery according to myopenia, Kaplan–Meier methods and log-rank test were used. Cox regression analysis was performed to evaluate the risk factors of surgery among CD patients treated with biological agents. A *p*-value of < 0.05 was considered statistically significant. Statistical analyses were performed using the R statistical software version 4.1.3 (R Foundation for Statistical Computing, Vienna, Austria).

## Results

### Patient characteristics

**Fig. 2:**
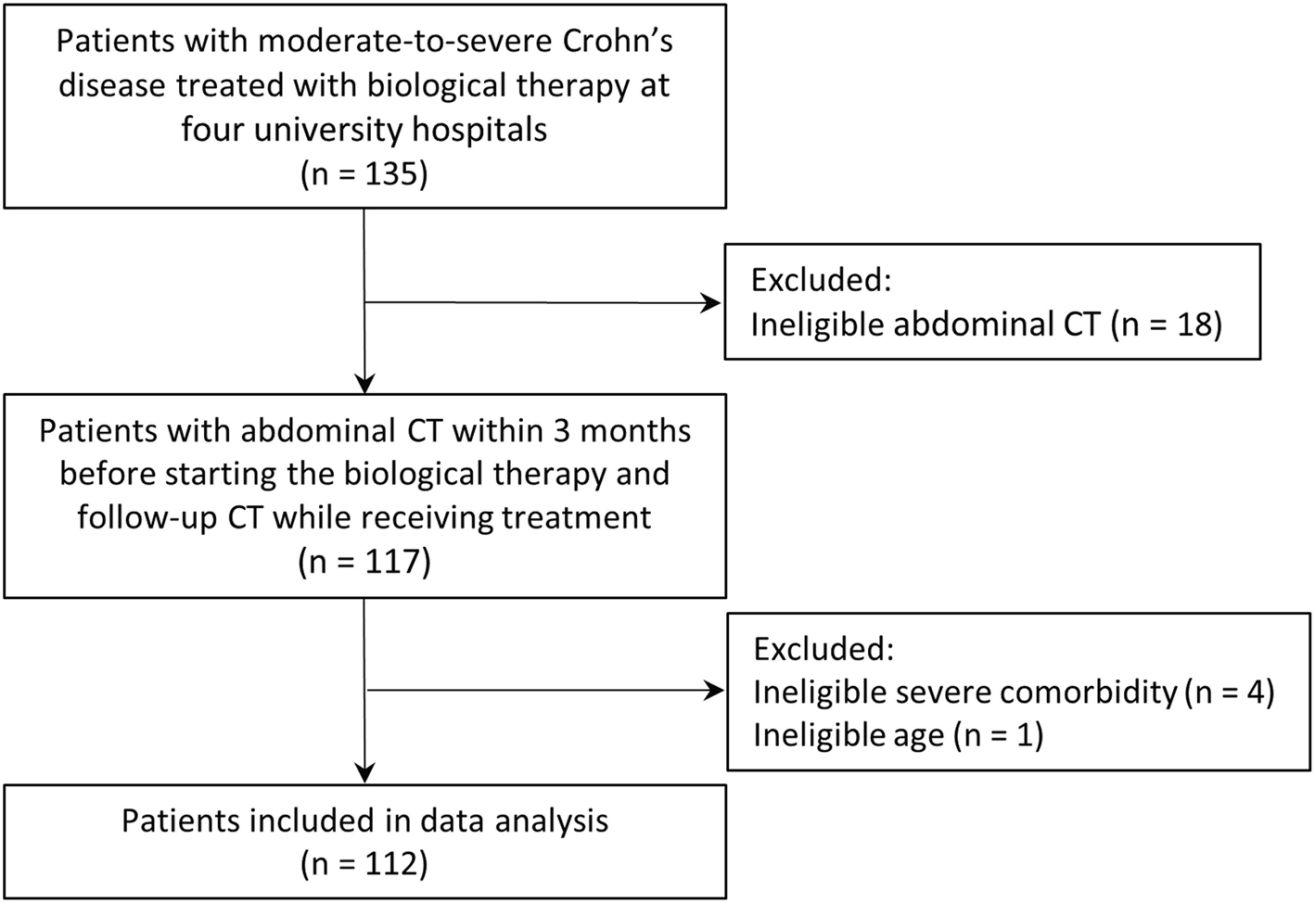
Flowchart of the study population

A total of 112 patients were included in this study (Fig. 2). The prevalence of myopenia was 70.5% (79 patients) according to Korean-specific cut-off values. The baseline characteristics of patients stratified according to the presence or absence of myopenia are summarized in Table 1. Myopenia was significantly associated with the male sex (*P* < 0.001) and BMI (*P* = 0.010). Altogether, 48 patients (42.9%) had previously undergone abdominal surgery. The location of the disease was the ileocolon in 66 patients (58.9%) and the stricturing type was the most common behavior of the disease in 49 patients (43.8%), followed by the inflammatory type in 42 patients (37.5%). The majority of patients were treated with IFX (67.9%), followed by ADA (40.2%), and the median duration of biological treatment was 64.0 months. There were no significant differences in the characteristics, except for sex and BMI, between the patients with and without myopenia.

**Table 1:** Baseline characteristics of patients with Crohn's disease

Characteristics	Total (*n* = 112)	Myopenia (*n* = 79)	Non-myopenia (*n* = 33)	*P*-value
Age (years)	35.5 (28.5–41.0)	36.0 (29.0–40.5)	32.0 (27.0–41.0)	0.293
Male sex	82 (73.2%)	68 (86.1%)	14 (42.4%)	< 0.001
BMI (kg/m^2^)	21.0 (18.5–23.7)	20.6 (17.7–22.8)	22.3 (20.1–24.5)	0.010
Previous abdominal surgery	48 (42.9%)	34 (43.0%)	14 (42.4%)	1.000
Smoking habit	17 (15.2%)	15 (19.0%)	2 (6.1%)	0.147
Montreal classification				
Age at diagnosis (years)				
A2(18–40)	91 (81.2%)	65 (82.3%)	26 (78.8%)	0.868
A3(> 40)	21 (18.8%)	14 (17.7%)	7 (21.2%)	0.868
Location				
L1(Ileum)	20 (17.9%)	12 (15.2%)	8 (24.2%)	0.384
L2(Colon)	26 (23.2%)	21 (26.6%)	5 (15.2%)	0.289
L3(Ileocolon)	66 (58.9%)	46 (58.2%)	20 (60.6%)	0.982
L4(Upper GI)	18 (16.1%)	12 (15.2%)	6 (18.2%)	0.912
Behavior				
B1(Inflammatory)	42 (37.5%)	30 (38.0%)	12 (36.4%)	1.000
B2(Stricturing)	49 (43.8%)	33 (41.8%)	16 (48.5%)	0.657
B3(Penetrating)	21 (18.8%)	16 (20.3%)	5 (15.2%)	0.715
P(Perianal disease)	70 (62.5%)	49 (62.0%)	21 (63.6%)	1.000
Extra-intestinal manifestation	44 (39.3%)	30 (38.0%)	14 (42.4%)	0.820
Disease duration (years)	10.9 (7.2–14.4)	11.0 (7.6–14.6)	9.2 (6.8–13.8)	0.264
Biologics				
Infliximab	76 (67.9%)	51 (64.6%)	25 (75.8%)	0.350
Adalimumab	45 (40.2%)	33 (41.8%)	12 (36.4%)	0.748
Ustekinumab	6 (5.4%)	4 (5.1%)	2 (6.1%)	1.000
Vedolizumab	1 (0.9%)	0 (0.0%)	1 (3.0%)	0.651
Refractory (2 or more biologics)	13 (11.6%)	7 (8.9%)	6 (18.2%)	0.280
Biologics treatment duration (months)	64.0 (30.5–93.5)	66.0 (32.0–97.5)	58.0 (26.0–82.0)	0.457
Operation after biologics	21 (18.8%)	18 (22.8%)	3 (9.1%)	0.154
Concurrent medical treatment				
steroid	25 (22.3%)	16 (20.3%)	9 (27.3%)	0.572
5-ASA	94 (83.9%)	65 (82.3%)	29 (87.9%)	0.650
Azathioprine	56 (50.0%)	40 (50.6%)	16 (48.5%)	1.000

Values are presented as median (Interquartile range) or number (%)

BMI, body mass index; 5-ASA, 5-aminosalicylic acid

The serum albumin level was significantly associated with myopenia (3.7 vs. 4.0 mg/dL, *P* = 0.005). The body composition values were measured based on CT before the initiation of the biologic treatment and are presented in Table S1. The median SMI was 37.7 and 44.8 cm^2^/m^2^ in the myopenia and non-myopenia groups, respectively (*P* = 0.001). The SFA (44.3 vs. 90.4 cm^2^/m^2^, *P* < 0.001), VFA (26.1 vs. 33.8 cm^2^/m^2^, *P* = 0.039), and TFA (86.1 c vs. 156.5 cm^2^/m^2^, *P* < 0.001) were significantly lower in the myopenia group than in the non-myopenia group. The median duration between baseline and follow-up CT was 4.1 years. All patients in our study were continuously treated with biologics between the initial assessment and the follow-up CT.

### Comparison of the laboratory and body composition values before and after biological therapy

Significant changes in laboratory values were observed after biological therapy, which includes a decrease in CRP levels (1.46 vs. 0.27 mg/dL, *P* < 0.001) and an increase in albumin (3.80 vs. 4.37 mg/dL, *P* < 0.001) and hemoglobin (12.15 vs. 13.35 mg/dL, *P* < 0.001) levels (Table S2).

All body composition parameters were significantly increased after treatment with biological agents. The median SMI was 38.1 cm^2^/m^2^ and 39.80 cm^2^/m^2^ at baseline and after biologic therapy (*P* < 0.001), respectively. The SFA (54.61 vs. 93.20 cm^2^/m^2^, *P* < 0.001), VFA (28.98 vs. 53.15 cm^2^/m^2^, *P* < 0.001), and TFA (94.40 vs. 173.32 cm^2^/m^2^, *P* < 0.001) were significantly increased after treatment with biologics.

**Fig. 3:**
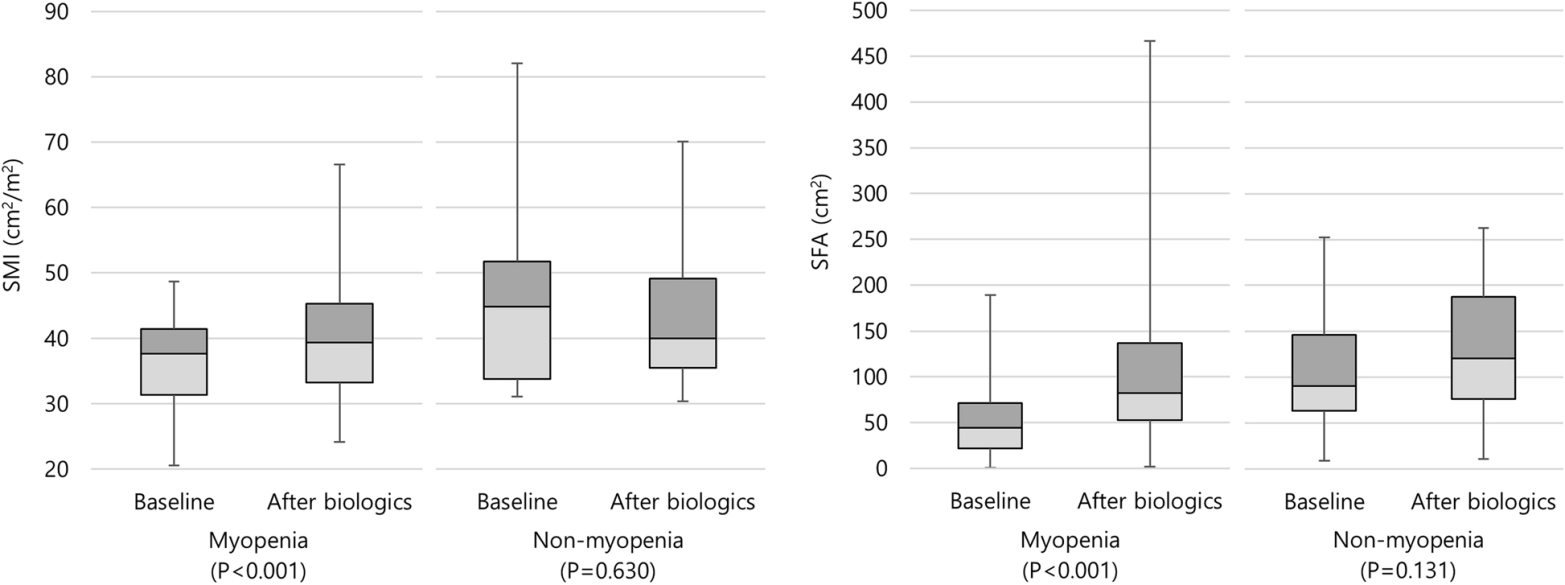
The changes in SMI and SFA at baseline and after biologic treatment for Crohn’s disease patients in the myopenia and non-myopenia

The results of the subgroup analysis by myopenia are shown in Tables 2 and S3. In the myopenia group, all values of laboratory and body composition on CT were significantly different before and after the biologic treatment (Table 2). However, in the patient without myopenia, no significant differences were found in the body composition parameter values at baseline and post-biological therapy (Table S3). Figure 3 shows the comparison of SMI and SFA before and after the use of biological agents in the myopenia and non-myopenia.

**Table 2:** Laboratory and body composition parameter values at baseline and after biologic treatment for Crohn's disease patients with myopenia

	Baseline	Post biologics	*P*-value
Laboratory parameters			
CRP (mg/dL)	1.63 (0.58–4.40)	0.26 (0.06–1.67)	0.004
Hemoglobin (mg/dL)	12.20 (10.50–13.68)	13.60 (11.43–14.78)	0.002
Albumin (mg/dL)	3.70 (3.30–4.10)	4.30 (3.89–4.59)	< 0.001
CT parameter value			
SMA (cm^2^)	110.86 (93.45–122.57)	118.50 (96.18–136.83)	< 0.001
SMI (cm^2^/m^2^)	37.68 (31.40–41.48)	39.40 (33.29–45.35)	< 0.001
PMA (cm^2^)	17.81 (12.35–22.97)	17.93 (13.51–25.60)	0.003
SFA (cm^2^)	44.29 (21.62–71.61)	82.42 (51.88–137.10)	< 0.001
VFA (cm^2^)	26.12 (13.55–43.35)	54.61 (32.04–89.87)	< 0.001
TFA (cm^2^)	86.11 (41.52–137.24)	165.36 (103.34–263.28)	< 0.001
IMFA (cm^2^)	5.42 (2.52–19.97)	10.43 (5.48–29.49)	< 0.001

Values are presented as median (interquartile range)

*CRP* C-reactive protein, *SMA* Skeletal muscle area, *SMI* Skeletal muscle index, *PMA* Psoas muscle area, *SFA* Subcutaneous fat area, *VFA* Visceral fat area, *TFA* Total fat area, *IMFA* Intramuscular fat area

### Association between SMI and clinical variables

The correlations between SMI and clinical variables in patients with CD are shown in Table 3. The BMI (rho = 0.437), PMA (rho = 0.672), Albumin (rho = 0.285), and hemoglobin (r = 0.282) showed an increasing trend along with increasing SMI. The BMI was significantly correlated with SFA (rho = 0.548) and TFA (rho = 0.539).

**Table 3:** Correlation between SMI and clinical parameters

	BMI	Height	Age	SFA	VFA	PMA	TFA	IMFA	Albumin	CRP	Hb
SMI	0.437^b^	0.180	− 0.076	0.128	0.137	0.672^b^	0.111	− 0.083	0.285^b^	− 0.194^a^	0.282^b^
BMI		0.098	− 0.159	0.548^b^	0.447^b^	0.318^b^	0.539^b^	0.288^b^	0.180	− 0.084	0.195^a^
Height			− 0.016	− 0.186^a^	− 0.012	0.514^b^	− 0.097	0.151	− 0.151	0.152	0.201^a^
Age				− 0.078	0.151	− 0.110	− 0.006	− 0.010	− 0.071	− 0.108	− 0.109
SFA					0.698^b^	0.027	0.932^b^	0.364^b^	0.222^a^	− 0.212^a^	− 0.103
VFA						0.081	0.857^b^	0.466^b^	− 0.004	− 0.082	− 0.141
PMA							0.101	0.260^b^	0.095	− 0.051	0.319^b^
TFA								0.546^b^	0.105	− 0.163	− 0.117
IMFA									− 0.252^b^	0.176	− 0.072
Albumin										− 0.309^b^	0.487^b^
CRP											− 0.114

^a^Correlation is significant at the 0.05 level

^b^Correlation is significant at the 0.01 level

*SMI* Skeletal muscle index, *BMI* Body mass index, *SFA* Subcutaneous fat area, *VFA* Visceral fat area, *PMA* Psoas muscle area, *TFA* Total fat area, *IMFA* Intramuscular fat area, *CRP* C-reactive protein, *Hb* Hemoglobin

### Predictors of surgery in patients with myopenia

**Fig. 4:**
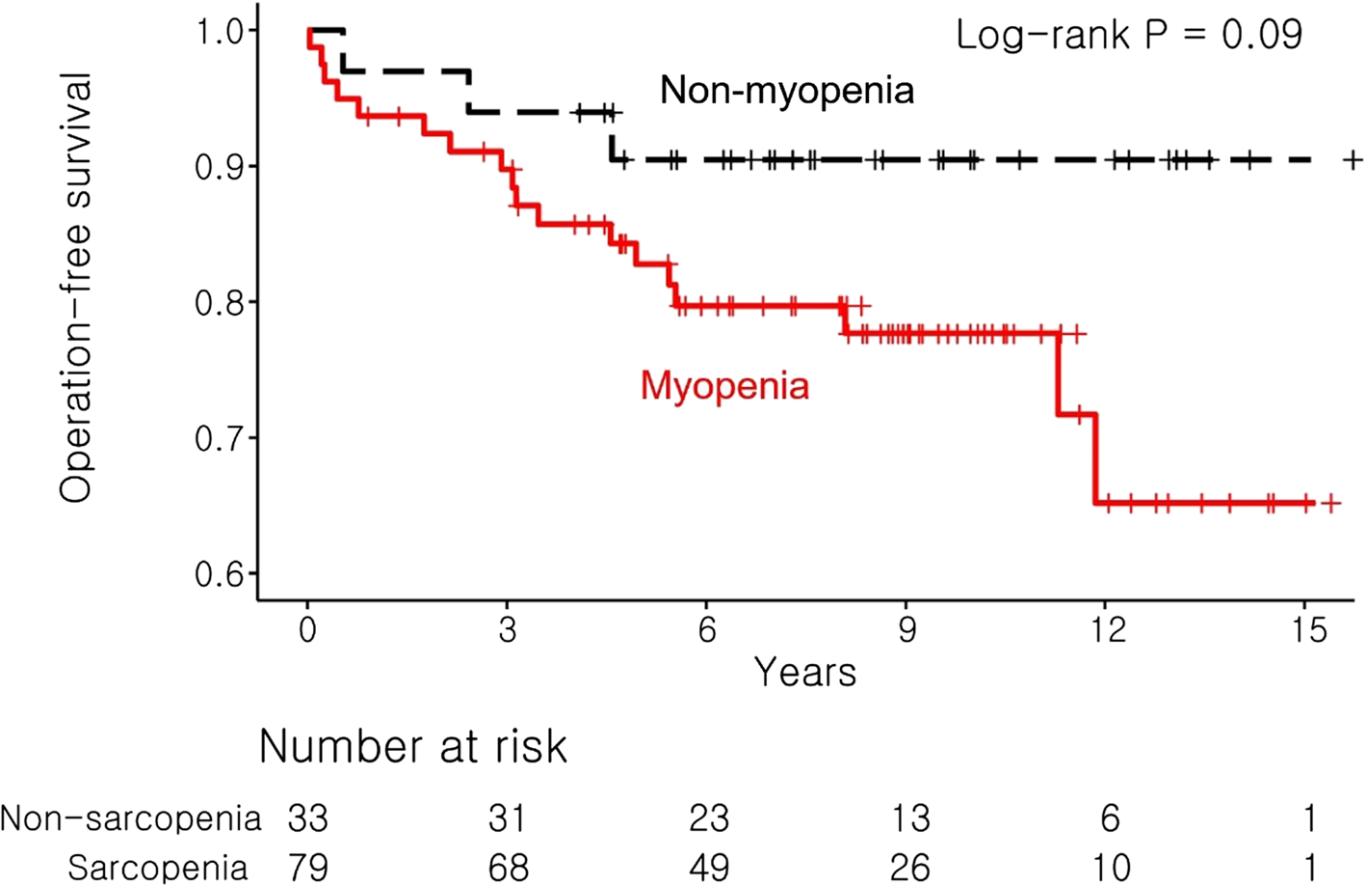
Operation-free survival rate by Kaplan–Meier analysis for Crohn's disease patients with or without myopenia

The results of the Kaplan–Meier analysis for CD patients showed that the operation-free survival rate tended to decrease in patients with myopenia, as compared to that of patients without myopenia (Log-rank test, *P* = 0.090; Fig. 4). The result of the univariable Cox regression analysis showed that sex (hazard ratio [HR], 8.859; *P* = 0.034) and disease location at the ileocolon (HR, 0.338; *P* = 0.046) were significantly associated with operation-free survival. Contrarily, myopenia was not significantly related to operation-free survival (HR, 2.759; *P* = 0.104). In the multivariable analysis, sex (HR, 17.217; *P* = 0.021) and penetrating CD (HR, 5.399; *P* = 0.020) were independent predictive factors of surgery (Table 4).

**Table 4:** Cox regression hazard ratio in the univariate and multivariate analyses for surgery in patients with Crohn’s disease

Variable	Univariable		Multivariable	
	HR (95% CI)	*P*-value	HR (95% CI)	*P*-value
Myopenia	2.759 (0.812–9.375)	0.104	0.739 (0.185–2.948)	0.668
Male sex	8.859 (1.182–66.4)	0.034	17.217 (1.542–192.250)	0.021
Age	0.975 (0.927–1.026)	0.329		
BMI	0.997 (0.884–1.124)	0.961		
Age at diagnosis (years)				
A2 (18–40)	Ref			
A3 (> 40)	0.963 (0.321–2.893)	0.946		
Location				
L1(Ileum)	Ref		Ref	
L2(Colon)	0.697 (0.233–2.087)	0.519	1.279 (0.390–4.194)	0.684
L3(Ileocolon)	0.338 (0.117–0.979)	0.046	0.464 (0.144–1.494)	0.198
L4(Upper GI)	0.947 (0.698–1.286)	0.729		
Behavior				
B1(Inflammatory)	Ref		Ref	
B2(Stricturing)	2.056 (0.797–7.878)	0.116	1.709 (0.483–6.051)	0.406
B3(Penetrating)	3.395 (0.956–12.056)	0.059	5.399 (1.311–22.241)	0.020
P(Perianal disease)	0.482 (0.205–1.136)	0.095	0.396 (0.150–1.047)	0.062
Disease duration	1.039 (0.960–1.125)	0.344		
Biologics				
Biologics one	Ref			
Biologics 2 or more	1.157 (0.337–3.974)	0.817		
Biologics duration	1.003 (0.993–1.014)	0.554		
Laboratory parameters				
Hemoglobin	0.950 (0.770–1.173)	0.636		
Albumin	0.736 (0.355–1.527)	0.411		
CRP	0.940 (0.838–1.054)	0.288		
CT parameter value				
SMA	1.005 (0.992–1.017)	0.466		
PMA	1.027 (0.972–1.085)	0.342		
SFA	0.991 (0.981–1.001)	0.075	0.963 (0.777–1.193)	0.731
VFA	0.985 (0.966–1.003)	0.103	0.934 (0.754–1.155)	0.527
TFA	0.994 (0.987–1.000)	0.053	1.044 (0.844–1.292)	0.693
IMFA	0.973 (0.935–1.012)	0.166	0.952 (0.767–1.182)	0.656

*CRP* C-reactive protein, *SMA* Skeletal muscle area, *PMA* Psoas muscle area, *SFA* subcutaneous fat area, *VFA* Visceral fat area, *TFA* Total fat area, *IMFA* Intramuscular fat area, *HR* Hazard ratio

## Discussion

The present study evaluated the body composition changes in patients with CD before and after the use of biological agents. Several key observations were noted. First, the skeletal muscle and body fat in CD patients with myopenia were significantly increased after the administration of biologics. In particular, the increase in body fat was notable. Second, the BMI and albumin levels showed tendencies to increase along with an increase in skeletal muscle mass. Third, CD patients with myopenia may be at a higher risk for surgery than those without myopenia.

Biologics brought a paradigm shift in ways to treat CD. Inflammation suppressed through biological agents might contribute to an increase in muscle mass, which again suppress inflammation, thereby forming a virtuous cycle. Considering that in Crohn's disease, muscle loss may continue due to inflammation, [22] it is thought that the slight increase in muscle mass after the biological treatment is meaningful despite of median rather long duration of biological treatment. In this study, most of the patients received anti-TNF, and this increase in body composition may be caused by a direct effect on anti-TNF. Therefore, these results may differ from patients using another biological agents.

On the other hand, the increase in fat mass left us with another problem. In this study, the increase in fat mass was relatively higher than that in muscle mass after administration of the biologic agent. In CD, creeping fat, characterized by localized fat accumulation near the intestinal wall, induces intestinal inflammation. [23] Moreover, improvements in nutritional status may also negatively affect the patients, as this may result in metabolic syndrome. After successful treatment with biological agents in patients with CD, it might be necessary to modify their sedentary lifestyle and diet, such as establishing a healthy diet to prevent overweight. [24, 25]

Recent studies showed that albumin and BMI are associated with body composition parameters. [26, 27] In this study, BMI and albumin showed a possible correlation with skeletal muscle measurement. Therefore, BMI and albumin might be possible alternative indicators for estimating myopenia in patients who had not undergone an imaging study, but more research is needed to confirm this.

Although CD can be treated conservatively, surgery is required for cases with free perforation with peritonitis and massive hemorrhage, acute severe colitis, complete bowel obstruction, or bowel ischemia. [28] Myopenia was an independent predictor of surgery according to a study by Adrienn Erős et al. [29] Myopenia tended to increase the risk of surgery in CD patients in this study. Increasing muscle mass in CD patients can reduce the need for surgical intervention and postoperative complications. [2] Besides myopenia, the only independent predictor of surgery analyzed in this study was the penetrating type CD according to the Montreal classification. [30] The penetrating type also was reported to be associated with an increased risk of recurrence after surgery. [31] This is thought to be due to the nature of fistulas and abscesses which can develop. The predictive factors for surgery in CD patients may help in making treatment decisions, such as the type of drug and the timing of its introduction.

The cutoff values used to define the sarcopenia are applied depending on the study and remain controversial. Considering that there is a difference in muscle mass according to race in previous studies, the threshold should be set differently according to race. Zhang et al. defined sarcopenia as an SMI < 49.9 cm^2^/m^2^ for men and < 28.7 cm^2^/m^2^ for women, which is a new cut-off value of sarcopenia determined specifically for the Asian population. [32] Because the physical characteristics are different in the case of Asians. CT-based studies in Korean patients also vary from the cutoffs presented. [33, 34] We defined sarcopenia by Korean specific cut-off values for the SMI of 49 cm^2^/m^2^ for men and 31 cm^2^/m^2^ for women, which was used in several previous studies which included Korean population. [21, 35] Additional research is needed to consensus on the definition of sarcopenia in the future.

This study has a few limitations. First, there was an imbalance between the male and female ratios. It is known that the prevalence of CD in women is high in Europe and the United States, but the opposite result was observed in Asian patients. [36] In this study, the proportion of men was 73.2%. There is a difference in the degree of muscle formation and muscle mass between men and women, and this needs to be considered. Second, the effect of concurrent medications, such as steroids and 5-aminosalicylic acid, on myopenia was not considered. Corticosteroids, used as first-line treatments for CD, are effective in inducing remission and reducing inflammation. [37] Although corticosteroids may reduce skeletal muscle mass and strength with a different mechanism of action from biological agents, it is thought that they may have a role in contributing to sarcopenia in terms of reducing inflammation, and thus additional research is needed. In addition to the effects of medications, it is also necessary to consider the effects of diet and physical activity during the period of therapy. Third, the number of enrolled CD patients without myopenia was too small, limiting the comparison between myopenia and non-myopenia patients. According to a previous study, approximately 50% of CD patients had myopenia, whereas, in this study, 70.5% had myopenia [1, 20]. This is because biologics are used when the first-line treatment is not effective, and the patients in our study are more likely to have advanced diseases. Finally, there is a selection bias because it is difficult to accurately determine the indications for which CT was performed due to a retrospective multicenter design. Due to several confounding factors, the cohort examined may be atypical. Therefore, the results should be clarified later through more prospective studies.

## Conclusions

This study revealed that biological agents can increase all body composition parameters among CD patients with myopenia. Anti-TNF may reverse cachexia in CD patients, allowing them to survive without surgery.

## Supplementary Information

**Additional file 1: Supplementary Table S1.** Laboratory and bodycomposition parameter values at baseline in patients with Crohn's disease.**Table S2.** Laboratory and body composition parameter values at baseline andafter biologic treatment for Crohn'sdisease patients. **Table S3.** Laboratory and body composition parametervalues at baseline and after biologic treatment for Crohn's disease patientswithout myopenia.

## Data Availability

The de-identified participant data that support the findings of this study are available upon reasonable request to the corresponding author.

## Abbreviations

BMI: Body mass index
CD: Crohn’s disease
CRP: C-reactive protein
CT: Computed tomography
HR: Hazard ratio
IBD: Inflammatory bowel disease
IMFA: Intramuscular fat area
IRB: Institutional Review Board
SFA: Subcutaneous fat area
SMA: Skeletal muscle area
SMI: Skeletal muscle index
TFA: Total fat area
VFA: Visceral fat area
