# Supplementary material for: The effect of biological agent on body composition in patients with Crohn’s disease
Source: BMC Gastroenterol. 2023 Mar 30;23:100. doi: 10.1186/s12876-023-02742-2 (PMC10064761; doi:10.1186/s12876-023-02742-2)
Supplement: Supplementary file 1 — Additional file 1: Supplementary Table S1. Laboratory and bodycomposition parameter values at baseline in patients with Crohn's disease.Table S2. Laboratory and body composition parameter values at baseline andafter biologic treatment for Crohn'sdisease patients. Table S3. Laboratory and body composition parametervalues at baseline and after biologic treatment for Crohn's disease patientswithout myopenia. [file 12876_2023_2742_MOESM1_ESM.docx]

**Supplementary Materials:** Supplementary Table S1: Laboratory and body composition parameter values at baseline in patients with Crohn's disease. Table S2: Laboratory and body composition parameter values at baseline and after biologic treatment for Crohn's disease patients. Table S3: Laboratory and body composition parameter values at baseline and after biologic treatment for Crohn's disease patients without myopenia.

| Table S1. Laboratory and body composition parameter values at baseline in patients with Crohn's disease. | | | | | | | | |
| --- | --- | --- | --- | --- | --- | --- | --- | --- |
|  |  | |  | |  | |  | |
|  | Total  (*n* = 112) | | Myopenia (*n* = 79) | | Non-myopenia (*n* = 33) | | *P*-value | |
| Laboratory parameters |  | |  | |  | |  | |
| CRP (mg/dL) | 1.5 (0.5–4.0) | | 1.6 (0.6–4.3) | | 1.3 (0.3–2.7) | | 0.216 | |
| Hemoglobin (mg/dL) | 12.1 (10.5–13.5) | | 12.2(10.5–13.6) | | 12.1(10.7–13.0) | | 0.519 | |
| Albumin (mg/dL) | 3.8 (3.4–4.2) | | 3.7 (3.3–4.1) | | 4.0 (3.7–4.3) | | 0.005 | |
|  |  | |  | |  | |  | |
| CT parameter value |  | |  | |  | |  | |
| SMA (cm^2^) | 111.3 (89.2–127.7) | | 110.9 (93.7–122.4) | | 111.6 (84.3–153.8) | | 0.202 | |
| SMI (cm^2^/m^2^) | 38.2 (32.4–44.5) | | 37.7 (31.4–41.5) | | 44.8 (33.8–51.7) | | 0.001 | |
| PMA (cm^2^) | 17.8 (11.8–24.4) | | 17.8 (12.5–22.9) | | 17.3 (10.5–26.5) | | 0.414 | |
| SFA (cm^2^) | 54.6 (26.6–97.2) | | 44.3 (22.0–71.1) | | 90.4 (63.3–146.6) | | <0.001 | |
| VFA (cm^2^) | 28.9 (16.1–49.7) | | 26.1 (14.1–43.2) | | 33.8 (19.2–68.0) | | 0.039 | |
| TFA (cm^2^) | 94.4 (60.9–172.4) | | 86.1 (41.7–136.4) | | 156.5 (89.3–284.3) | | <0.001 | |
| IMFA (cm^2^) | 5.6 (2.7–16.2) | | 5.4 (2.5–18.7) | | 6.4 (3.0–11.7) | | 0.628 | |
|  |  | |  | |  | |  | |
| Duration of follow-up CT (years) | 4.1 (1.8–7.4) | | 4.2 (2.0–7.5) | | 4.1(1.7–6.4) | | 0.846 | |
| Values are presented as median (interquartile range) | | | | | | | | |
| CRP, C-reactive protein; SMA, skeletal muscle area; SMI, skeletal muscle index; PMA, psoas muscle area; SFA, subcutaneous fat area; VFA, visceral fat area; TFA, total fat area; IMFA, intramuscular fat area; CT, computed tomography | | | | | | | | |
|  |  |  |  |  |  |  |  |  |
|  |  |  |  |  |  |  |  |  |
| Table S2. Laboratory and body composition parameter values at baseline and after biologic treatment for Crohn's disease patients. | | | | | | | |  |
|  | |  | |  | |  | |  |
|  | | Baseline | | Post biologics | | *P*-value | |  |
| Laboratory parameters | | | |  | |  | |  |
| CRP (mg/dL) | | 1.46 (0.50–4.04) | | 0.27 (0.09–1.45) | | <0.001 | |  |
| Hemoglobin (mg/dL) | | 12.15 (10.50–13.50) | | 13.35 (11.55–14.50) | | <0.001 | |  |
| Albumin (mg/dL) | | 3.80 (3.40–4.25) | | 4.37 (4.02–4.62) | | <0.001 | |  |
|  | |  | |  | |  | |  |
| CT parameter value | |  | |  | |  | |  |
| SMA (cm^2^) | | 111.25 (89.20–127.75) | | 117.88 (93.68–139.52) | | <0.001 | |  |
| SMI (cm^2^/m^2^) | | 38.19 (32.41–44.55) | | 39.80 (33.66–46.73) | | <0.001 | |  |
| PMA (cm^2^) | | 17.80 (11.81–24.40) | | 18.14 (13.45–25.83) | | 0.018 | |  |
| SFA (cm^2^) | | 54.61 (26.65–97.21) | | 93.20 (60.00–148.97) | | <0.001 | |  |
| VFA (cm^2^) | | 28.98 (16.09–49.68) | | 53.15 (29.87–84.76) | | <0.001 | |  |
| TFA (cm^2^) | | 94.40 (60.86–172.41) | | 173.32 (107.00–267.53) | | <0.001 | |  |
| IMFA (cm^2^) | | 5.61 (2.66–16.15) | | 9.63 (4.52–23.62) | | <0.001 | |  |
| Values are presented as median (interquartile range) | | | | | | | |  |
| CRP, C-reactive protein; SMA, skeletal muscle area; SMI, skeletal muscle index; PMA, psoas muscle area; SFA, subcutaneous fat area; VFA, visceral fat area; TFA, total fat area; IMFA, intramuscular fat area | | | | | | | |  |
|  |  |  |  |  |  |  |  |  |

| Table S3. Laboratory and body composition parameter values at baseline and after biologic treatment for Crohn's disease patients without myopenia. | | | |
| --- | --- | --- | --- |
|  |  |  |  |
|  | Baseline | Post biologics | *P*-value |
| Laboratory parameters |  |  |  |
| CRP (mg/dL) | 1.30 (0.31–2.79) | 0.33 (0.18–1.12) | 0.011 |
| Hemoglobin (mg/dL) | 12.10 (10.63–13.05) | 12.60 (11.75–13.95) | 0.045 |
| Albumin (mg/dL) | 4.00 (3.68–4.33) | 4.50 (4.20–4.69) | 0.001 |
|  |  |  |  |
| CT parameter value |  |  |  |
| SMA (cm^2^) | 111.60 (84.18–154.78) | 115.10 (89.85–141.08) | 0.598 |
| SMI (cm^2^/m^2^) | 44.84 (33.73–51.98) | 40.01 (35.40–49.53) | 0.630 |
| PMA (cm^2^) | 17.30 (10.48–26.53) | 18.40 (13.15–27.30) | 0.893 |
| SFA (cm^2^) | 90.40 (63.23–158.68) | 120.50 (74.88–188.15) | 0.131 |
| VFA (cm^2^) | 33.80 (19.03–73.45) | 45.00 (28.85–68.78) | 0.131 |
| TFA (cm^2^) | 156.50 (87.38–284.93) | 187.10 (118.88–300.58) | 0.053 |
| IMFA (cm^2^) | 6.40 (2.98–12.85) | 7.00 (3.60–13.35) | 0.186 |
| Values are presented as median (interquartile range) | | | |
| CRP, C-reactive protein; SMA, skeletal muscle area; SMI, skeletal muscle index; PMA, psoas muscle area; SFA, subcutaneous fat area; VFA, visceral fat area; TFA, total fat area; IMFA, intramuscular fat area | | | |
|  |  |  |  |
